# The Significance of OCTA in Studying Vessel Density and Retinal Thickness in Individuals with Myopia

**DOI:** 10.3390/medicina61030532

**Published:** 2025-03-18

**Authors:** Marija Veselinović, Marija Trenkić, Vladimir Čanadanović, Predrag Jovanović, Aleksandar Veselinović, Maja Petrović, Aida Kasumović Bećirović

**Affiliations:** 1 Ophthalmology Clinic, University Clinical Center Niš, Boulevard Dr Zorana Đinđića 48, 18000 Niš, Serbia; marija.trenkic@gmail.com (M.T.); pedja.jovanovic64@gmail.com (P.J.); drpetrovicmaja@gmail.com (M.P.); 2 Faculty of Medicine, University of Niš, Boulevard Dr Zorana Đinđića 81, 18000 Niš, Serbia; 3 Eye Clinic, University Clinical Center of Vojvodina, Hajduk Veljkova 1–9, 21000 Novi Sad, Serbia; canadnovic.vladimir@gmail.com; 4 Faculty of Medicine, University of Novi Sad, Hajduk Veljkova 3, 21000 Novi Sad, Serbia; 5 Special Hospital for Ophthalmology “Veselinović”, Bulevar Nemanjića 67a, 18000 Niš, Serbia; veselinovicaleksandar@gmail.com; 6 Eye Polyclinic “Dr. Sefić”, Ferhadija 5, 71000 Sarajevo, Bosnia and Herzegovina; aidakasumovic1@hotmail.com

**Keywords:** OCTA, myopia, vessel density, retina

## Abstract

*Background and Objectives*: This study explores the relationship between retinal structure, vascular densities (VD), and the progression of myopia, aiming to identify novel biomarkers for assessing myopia severity. *Materials and Methods*: A total of 260 eyes were divided into four groups: Emmetropia (EM) (*n* = 74), Low Myopia (LM) (*n* = 68), Moderate Myopia (MM) (*n* = 64), and High Myopia (HM) (*n* = 54). VD and retinal thickness (RT) in the macular and peripheral quadrants were measured using optical coherence tomography-angiography (OCTA). SVD and DVD were analyzed across the paranasal, peritemporal, perisuperior, and peri-inferior quadrants. *Results*: Significant differences in superficial vessel density (SVD) were found in the paranasal (EM vs. MM, *p* = 0.017; EM vs. HM, *p* = 0.001), peritemporal (EM vs. MM, *p* = 0.006; EM vs. HM, *p* = 0.001; LM vs. HM, *p* = 0.004; MM vs. HM, *p* = 0.032), perisuperior (EM vs. MM, *p* = 0.005; EM vs. HM, *p* = 0.001; LM vs. HM, *p* = 0.027), and perifoveal quadrants (EM vs. MM, *p* = 0.003; EM vs. HM, *p* = 0.008; LM vs. HM, *p* = 0.004; MM vs. HM, *p* = 0.012). Deep vessel density (DVD) showed significant differences in the paranasal (*p* = 0.012–0.022), peritemporal (*p* = 0.002–0.026), perisuperior (*p* = 0.003–0.034), perifoveal (*p* = 0.002–0.017), and peri-inferior (*p* = 0.002–0.022) quadrants. Retinal thickness was significantly reduced in HM eyes, with the most pronounced reduction in the peritemporal quadrant (mean difference: 16.7 ± 3.2 µm; *p* < 0.001). *Conclusions*: Structural and vascular changes in the retina become more pronounced as myopia progresses from moderate to high. The strong correlation between DVD, RT, and myopia severity highlights their potential as reliable biomarkers for monitoring myopia progression through OCTA imaging. These findings provide new insights into the vascular and structural changes underlying myopia and their diagnostic significance.

## 1. Introduction

Myopia, commonly known as nearsightedness, is the most prevalent refractive error worldwide, affecting approximately 25% of the global adult population. Projections suggest that by 2050, nearly half of the world’s population will be myopic, raising significant public health concerns due to the increasing burden of myopia-related complications [1]. The rapid rise in axial myopia, particularly among younger populations, is primarily attributed to environmental and behavioral factors, including increased urbanization, intensive educational demands, prolonged near work, and reduced exposure to natural light [2,3]. The primary driver of myopia progression and its pathological complications is excessive elongation of the eye’s axial length (AL) [4,5], indicating that a substantial proportion of today’s young myopic individuals are at risk of developing irreversible vision impairment and structural eye damage later in life [2,3]. High myopia, defined as a spherical equivalent (SE) exceeding −6 diopters (D), is one of the leading causes of permanent visual impairment globally. The key pathophysiological mechanism is excessive axial elongation, which leads to chorioretinal stretching and thinning, resulting in progressive and often irreversible damage to the retinal structure [4,5,6]. This process increases the risk of severe complications, including macular holes, chorioretinal atrophy, optic nerve head deformation, posterior staphyloma, and myopic foveoschisis, all of which contribute to progressive vision loss and functional decline [7]. Crucially, these structural changes are closely tied to alterations in retinal microvasculature, underscoring the importance of early and precise evaluation of macular microvascular changes in highly myopic eyes to prevent or delay disease progression [8,9,10].

The advent of Optical Coherence Tomography (OCT) has significantly enhanced the ability to assess retinal structure with high resolution, transforming the diagnosis and management of myopia-related complications. Building on this technology, Optical Coherence Tomography Angiography (OCTA) provides non-invasive, high-resolution imaging of retinal and choroidal vasculature without the need for contrast agents [11,12,13]. OCTA detects changes in blood flow by capturing rapid, consecutive B-scans and analyzing light reflection and scattering on erythrocytes within blood vessels, generating a detailed vascular map of the retina and choroid. This method allows for the precise quantification of microvascular density and perfusion at different retinal levels, surpassing the capabilities of conventional imaging techniques [11,12,13]. OCTA has become an indispensable tool for assessing early microvascular alterations and monitoring disease progression in high myopia [14].

Recent studies have provided compelling evidence for the role of OCTA in evaluating retinal and choroidal microvasculature changes associated with myopia. Zheng et al. [4] reported a significant reduction in macular vessel density and choriocapillaris flow in highly myopic eyes, indicating compromised vascular integrity. Ucak et al. [6] found that deep vascular plexus (DVP) density decreases progressively with increasing axial length, particularly in the perifoveal and peripapillary regions, highlighting the impact of axial elongation on deep retinal perfusion. Similarly, Shi et al. [9] demonstrated that deep vessel density (DVD) is more susceptible to myopia-related changes than superficial vessel density (SVD), suggesting that microvascular remodeling primarily affects the deeper capillary networks. Furthermore, Jiang et al. [10] found that reduced retinal thickness in the macular and perifoveal regions correlates strongly with increased myopia severity, indicating that structural and vascular alterations are closely interconnected. These findings underscore the complex relationship between axial elongation, vascular density, and retinal structural remodeling in high myopia.

While previous studies have identified significant associations between myopia and changes in retinal vessel density and thickness, most have focused on global macular alterations rather than quadrant-specific changes. The relationship between deep vessel density (DVD), retinal thickness (RT), and myopia severity at the quadrant level remains poorly understood. Furthermore, the potential of DVD and RT as predictive biomarkers for monitoring myopia progression using OCTA has not been fully explored. A more detailed, quadrant-specific analysis is essential to clarify the mechanisms driving microvascular and structural remodeling in myopic eyes and to establish more accurate diagnostic and prognostic tools for myopia management.

This study aims to provide a comprehensive and detailed analysis of the quadrantal alterations in retinal capillaries and microstructure associated with increasing myopia severity. By focusing on both macular microcirculation and retinal thickness, this research seeks to identify novel biomarkers for assessing and monitoring myopia progression through OCTA imaging. These findings may enhance early diagnosis and improve the management of high myopia, contributing to the development of more targeted and effective therapeutic strategies for preventing myopia-related visual impairment.

## 2. Materials and Methods

### 2.1. Study Participants

Clinical data were collected from 260 eyes of 130 eligible participants at the Ophthalmologic Clinic, University Clinical Center Niš, Niš, Serbia, between 15 September 2020, and 1 March 2021. Each participant underwent a comprehensive ophthalmic evaluation conducted by experienced ophthalmologists, which included assessments of refraction, intraocular pressure (IOP), best corrected visual acuity (BCVA), visual field examination, dilated fundus examination, axial length (AL), and optical coherence tomography angiography (OCTA) using the RTVue XR Avanti AngioVue system (Optovue, Inc., Fremont, CA, USA). All evaluations were performed under standardized lighting and environmental conditions to minimize measurement variability. Participants aged 18 to 60 years with a BCVA of ≥1.0, IOP between 10 mmHg and 21 mmHg, and AL ranging from 21.5 mm to 29.00 mm were eligible for inclusion. Only participants without other ocular disorders apart from refractive errors were considered to ensure that the findings reflect changes specific to myopia progression. The classification of eyes into groups was based on spherical equivalent (SE) values and axial length according to the Expert Consensus on Prevention and Control of High Myopia (2023). Emmetropia (EM) was defined as SE between −0.75D and +0.75D (*n* = 74), low myopia (LM) as SE between −3.00D and −0.75D (*n* = 68), moderate myopia (MM) as SE between −6.00D and −3.00D (*n* = 64), and high myopia (HM) as SE ≤ −6.00D or AL ≥ 26.5 mm (*n* = 54). Classification was performed by two independent ophthalmologists, with discrepancies resolved through consensus to ensure consistency and accuracy in group assignment. Exclusion criteria were defined to eliminate potential confounding factors that could influence retinal microvasculature or structural integrity. Subjects with a history of neurological or systemic diseases (e.g., diabetes, hypertension), ocular trauma, intraocular surgery, or refractive procedures were excluded. Additionally, participants with recent medication use that could alter ocular blood flow, media opacity (e.g., cataract), or evidence of ocular inflammation or retinal disorders (e.g., diabetic retinopathy, glaucoma) were not considered. Poor image quality due to unstable fixation, severe astigmatism (>2.0 D), or motion artifacts also constituted exclusion criteria. To minimize selection bias, recruitment was conducted consecutively, and each participant was screened by two independent ophthalmologists to confirm adherence to the inclusion and exclusion criteria. A standardized imaging protocol was followed during OCTA acquisition to ensure consistency and reproducibility of the measurements.

### 2.2. Optical Coherence Tomography Angiography

After the ocular examinations, all participants underwent imaging conducted by a single operator (XY) using the RTVue XR Avanti Spectral Domain OCT system (Optovue, Inc., Fremont, CA, USA) equipped with AngioVue software (Version 2015.1.0.90). Motion Correction Technology (MCT) was employed to correct for eye movement during image acquisition, adjusting both horizontal and vertical scans [15]. The scan speed was set at 70,000 A-scans per second. Each OCTA scan consisted of an X-scan and a Y-scan, with each raster scan lasting approximately 2.9 s, resulting in a total scan time of about 6 s [16]. The imaging area was centered on the fovea with a field of view of 3 × 3 mm^2^, which corresponds to approximately 10°. RTVue software (Version 2015.1.0.71) was used to segment OCTA scans, measure parameters in the regions of interest, and separate microcirculation into the superficial capillary plexus (SCP) and deep capillary plexus (DCP), allowing for detailed layer-by-layer visualization. The SCP was defined as the region spanning from the internal limiting membrane to the outer border of the ganglion cell layer. The DCP was identified as the layer extending from the outer border of the SCP to the outer border of the outer plexiform layer. Vessel density (VD) was automatically calculated as the percentage of the total imaged area occupied by blood vessels, while retinal thickness (RT) was measured across the full retinal layer. Images were analyzed within three concentric regions centered on the fovea: the foveal region (1-mm radius), the parafoveal region (between 1-mm and 3-mm radius), and the perifoveal region (between 3-mm and 6-mm radius). The parafoveal and perifoveal regions were further divided into four quadrants: temporal, superior, nasal, and inferior [17]. Figures from the fovea and the four quadrants within the parafoveal and perifoveal regions were analyzed to assess differences across the four study groups. To ensure consistency and minimize variability, the imaging protocol involved acquiring three consecutive scans for each participant. Only the highest-quality scan, defined by a signal strength index (SSI) > 50 and absence of motion artifacts, was selected for analysis. Images with poor fixation, decentration, or a quality score below 7/10 were excluded. All scans were reviewed independently by two experienced ophthalmologists, and discrepancies in segmentation or quality assessment were resolved by consensus. In cases of borderline scan quality, manual refinement of segmentation and alignment was performed using the RTVue software to reduce measurement errors. Motion correction was applied using the integrated Motion Correction Technology (MCT) to reduce artifacts caused by involuntary eye movements. The AngioVue software algorithm automatically identified and segmented the superficial and deep vascular layers, with manual adjustments applied when necessary to improve accuracy. Vessel density and retinal thickness values were extracted separately for the SCP and DCP, enabling detailed regional analysis of microvascular density and structural changes. To further validate the accuracy and consistency of the OCTA measurements, inter- and intra-observer variability was assessed using intraclass correlation coefficients (ICCs). The ICC values for vessel density and retinal thickness were 0.92 and 0.95, respectively, indicating excellent reproducibility. In addition, Bland–Altman plots were constructed to assess the limits of agreement between repeated measurements, confirming the consistency and reliability of the data. A sensitivity analysis was conducted to evaluate the impact of borderline-quality scans on the final results, and no significant changes in the overall findings were observed. Artifact rejection and quality control were systematically performed throughout the imaging process. Scans with motion artifacts, low signal strength, segmentation errors, or poor fixation were automatically flagged by the software and manually reviewed by the ophthalmologists. The software’s automated artifact rejection algorithm was supplemented by manual assessment to ensure the exclusion of scans affected by blink artifacts or unstable fixation. Segmentation quality was reviewed for each scan, and realignment was performed when necessary to ensure accurate layer separation and minimize misclassification errors. To ensure consistent measurements across participants, the same device and software version were used for all imaging sessions. The imaging environment, including room lighting and participant positioning, was standardized to reduce external variability. The consistency of the segmentation was confirmed by comparing results across multiple sessions, and the repeatability of the measurements was evaluated by analyzing the coefficient of variation (CV). The CV values for vessel density and retinal thickness were 3.4% and 2.8%, respectively, confirming a high degree of measurement stability. All data were exported and analyzed using SPSS Statistics, Version 26.0 (IBM, Armonk, NY, USA). Automated data extraction and validation ensured that the results were free from transcription errors or misalignment issues. Data integrity was maintained through double-checking of the all values and independent verification by a third investigator to eliminate any potential inconsistencies.

### 2.3. Statistical Analysis

Continuous variables were tested for normality using the Shapiro–Wilk test and for variance homogeneity using the Levene test. Gender distribution among the groups was assessed using the Chi-square test. Differences between the groups were analyzed using one-way analysis of variance (ANOVA) with Bonferroni post hoc correction for pairwise comparisons to adjust for multiple testing and reduce the risk of Type I error. The Bonferroni correction was applied due to its conservative nature, which reduces the likelihood of false-positive results when performing multiple comparisons. Data are presented as mean ± Standard Error of the Mean (SEM) unless otherwise specified. Statistical significance was set at *p* < 0.05. All statistical analyses were performed using SPSS Statistics, Version 26.0 (IBM, Armonk, NY, USA). A sensitivity analysis was performed to assess the robustness of the findings, and subgroup analysis was conducted to account for potential confounding factors, including age, gender, and baseline axial length. The power of the study was calculated post hoc to confirm that the sample size was adequate to detect statistically significant differences in vessel density and retinal thickness across the study groups.

## 3. Results

This study enrolled a total of 93 patients diagnosed with myopia, comprising 48 men (51.62%) and 45 women (48.38%). The average age of participants in the myopia group was 32.65 ± 13.55, with women averaging 31.43 ± 14.25 and men 33.87 ± 12.85. There were no significant differences in age (*p* = 0.478) or sex distribution (χ^2^ = 2.75, df = 3, *p* = 0.654) across subjects. Subjects were categorized into three subgroups based on Spherical Equivalent (SE) values. The control group (CG) consisted of 37 individuals, including 20 men (54.05%) and 17 women (45.95%). The average age in the control group was 32.53 ± 13.43, with women averaging 34.65 ± 11.53 and men 30.41 ± 19.33. There were no age differences between men and women (*p* = 0.765). Values for SVD and DVD in the four diagnostic groups, with statistical analysis are presented in Table 1 and Table 2, respectively.

**Figure 1:**
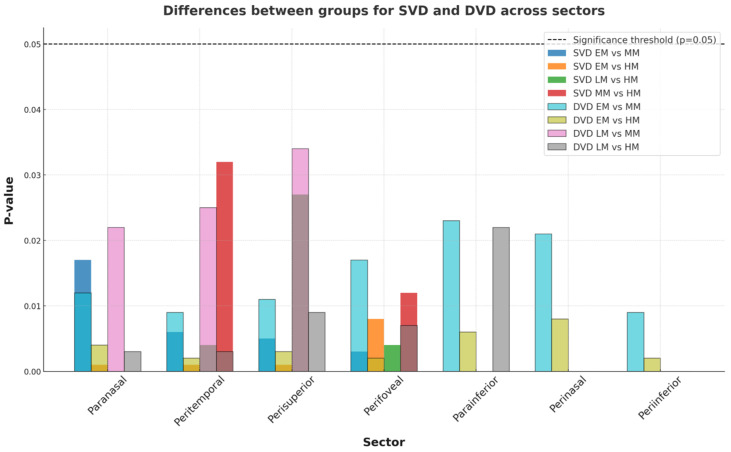
Differences in SVD and DVD between groups across retinal sectors.

In our study, superficial vessel density (SVD) showed a significant decrease across various sectors as emmetropia progressed. Specifically, reductions were observed in the paranasal sector between EM and MM (*p* = 0.017) and EM and HM (*p* = 0.001); the peritemporal sector between EM and MM (*p* = 0.006), EM and HM (*p* = 0.001), LM and HM (*p* = 0.004), and MM and HM (*p* = 0.032); the perisuperior sector between EM and MM (*p* = 0.005), EM and HM (*p* = 0.001), and LM and HM (*p* = 0.027); and the perifoveal sector between EM and MM (*p* = 0.003), EM and HM (*p* = 0.008), LM and HM (*p* = 0.004), and MM and HM (*p* = 0.012). Further, in our study deep vessel density (DVD) showed a significant decrease across various sectors as emmetropia progressed. Specifically, reductions were observed in the paranasal sector between EM and MM (*p* = 0.012), EM and HM (*p* = 0.004), LM and MM (*p* = 0.022) and LM and HM (*p* = 0.003); the parainferior sector between EM and MM (*p* = 0.023), EM and HM (*p* = 0.006) and MM and HM (*p* = 0.032); the perifovea sector between EM and MM (*p* = 0.017), EM and HM (*p* = 0.002) and LM and HM (*p* = 0.007); the peritemporal sector between EM and MM (*p* = 0.009), EM and HM (*p* = 0.002), LM and MM (*p* = 0.025), LM and HM (*p* = 0.003) and MM and HM (*p* = 0.026); the perisuperior sector between EM and MM (*p* = 0.011), EM and HM (*p* = 0.003), LM and MM (*p* = 0.034) and LM and HM (*p* = 0.009); the perinasal sector between EM and MM (*p* = 0.021) and EM and HM (*p* = 0.008); the peri-inferior sector between EM and MM (*p* = 0.009), EM and HM (*p* = 0.002), LM and HM (*p* = 0.022) and MM and HM (*p* = 0.022). Graphical presentation of the obtained results is given in Figure 1.

Our study demonstrated that retinal thickness, both superficial and deep, decreases with increasing myopia, Table 3 and Table 4. Regarding superficial retinal thickness, no significant decrease was observed between the MM and HM groups in any sector. Between the EM and LM groups, no significant decrease was found in the parafoveal, paratemporal, and paranasal sectors. Between the LM and MM groups, no significant decrease was noted in the parafoveal, paratemporal, paranasal, parainferior, and perisuperior sectors. For deep retinal thickness, a similar pattern was observed. Between the EM and LM groups, no significant decrease was determined in the parafoveal, paratemporal, parainferior, perifoveal, peritemporal, and perisuperior sectors. Between the EM and HM groups, no significant decrease was found only in the paranasal sector. Between the LM and MM groups, no significant decrease was identified in the parafoveal, paranasal, perinasal, and peri-inferior sectors. Between the LM and HM groups, no significant decrease was noted in the paranasal sector. However, between the MM and HM groups, significant decreases were observed in the paratemporal (*p* = 0.022), perinasal (*p* = 0.004), and peri-inferior (*p* = 0.012) sectors. These results indicate a consistent reduction in retinal thickness with myopia progression, though the significance varies depending on the sector and the diagnostic group.

## 4. Discussion

The global prevalence of high myopia is rapidly increasing, with projections indicating that nearly half of the world’s population will be myopic by 2050 [1]. This alarming trend has raised significant public health concerns due to the strong association between high myopia and vision-threatening complications. Severe myopia is linked to a higher risk of developing conditions such as myopic maculopathy, glaucoma, chorioretinal atrophy, and retinal detachment, which can result in irreversible visual impairment [18]. Highly myopic eyes, characterized by increased refractive error and axial length, are particularly prone to fundus vascular complications such as macular hemorrhage and choroidal neovascularization [19,20]. A considerable proportion of individuals with high myopia may progress to pathological myopia (PM), a more severe state marked by structural and vascular remodeling of the retina and choroid, highlighting the importance of early diagnosis and monitoring to mitigate long-term visual impairment [21].

OCT angiography (OCTA), a relatively recent advancement in ophthalmic imaging, has revolutionized the assessment of retinal microvasculature by providing high-resolution, non-invasive visualization of blood flow in the superficial and deep capillary plexuses. Unlike traditional fundus imaging, OCTA allows for precise quantification of vessel density and blood flow at different retinal depths, providing a detailed view of microvascular changes that were previously undetectable [2,22]. In this study, OCTA was employed to measure alterations in vessel density and retinal thickness across different regions of the retina in myopic eyes compared to emmetropic controls. The ability to analyze both superficial and deep vascular layers, as well as quadrant-specific changes, offers a more comprehensive understanding of the structural and vascular remodeling associated with myopia progression. While previous OCTA studies have primarily focused on the posterior pole of the eye, the complex dilation and elongation patterns in highly myopic eyes necessitate a broader evaluation of fundus changes across multiple regions, including the macula, perifovea, and parafovea [23,24].

Our results demonstrated a consistent and significant reduction in vessel density in highly myopic eyes, particularly within the deep capillary plexus (DCP). Vessel density in the perifoveal and parafoveal regions was significantly lower in high myopia compared to low and moderate myopia, suggesting that the DCP is more vulnerable to axial elongation and structural remodeling. The reduction in vessel density reflects compromised retinal perfusion, which may contribute to progressive choroidal and retinal thinning observed in pathological myopia [25]. The global elongation associated with high myopia likely exerts mechanical stress on the sclera, choroid, and retina, disrupting the microvascular network and leading to capillary dropout and ischemia. This is consistent with previous reports of reduced blood flow in the retrobulbar and retinal microvasculature in highly myopic eyes and the identification of larger non-perfused zones in the far peripheral retina [10,26].

The reduction in deep vessel density was most pronounced in the inferior, superior, and nasal parafoveal quadrants, suggesting that certain regions of the retina are more susceptible to axial elongation and vascular remodeling. High myopia has been linked to reduced retinal thickness and inner retinal layer dysfunction, which may contribute to decreased microvessel density due to lower metabolic demand or direct mechanical disruption of the capillary network [27,28,29,30,31]. Retinal vessel caliber narrowing in highly myopic eyes may further exacerbate the reduction in vessel density by limiting blood flow and increasing vascular resistance. The fact that DVD reduction was observed in specific quadrants rather than uniformly across the retina implies that myopia-related vascular remodeling follows a region-specific pattern influenced by localized mechanical and metabolic factors.

Interestingly, superficial vessel density (SVD) remained relatively stable across most quadrants during myopia progression, with significant reductions primarily observed in the paranasal, peritemporal, perisuperior, and peri-inferior sectors. This finding aligns with the study by Guo et al., who reported reduced SVD in the inferior and nasal quadrants in cases of high myopia [32]. The stability of SVD may reflect the greater resilience of the superficial vascular network, which is supplied by the central retinal artery and maintained by robust autoregulatory mechanisms. The SCP, which originates from the central retinal artery, relies on intrinsic autoregulatory mechanisms to maintain vascular function and meet metabolic demands. This mechanism may explain why SVD remains unchanged in most quadrants despite axial growth. However, the localized reduction in SVD in specific quadrants indicates that certain regions of the retina are more vulnerable to the mechanical and metabolic stress imposed by axial elongation [33].

The more pronounced reduction in DVD compared to SVD reinforces the theory that the deep vascular network is more susceptible to the mechanical stretching forces associated with axial elongation. The DCP plays a vital role in oxygen supplementation to the inner segment of photoreceptors and serves as the primary site for venous drainage of the retinal capillary plexus [34,35]. A progressive decline in DVD could disrupt venous drainage, leading to ischemia and further retinal thinning in highly myopic eyes. This hypothesis is supported by the work of Yao et al., who found that microvascular densities in various sectors of the macular area were significantly lower in high myopia compared to low and moderate myopia [36]. Yao et al. also suggested that reduced production of vascular endothelial growth factor (VEGF) due to retinal thinning may contribute to the decline in vessel density. Retinal thinning during myopia progression may lead to degeneration of retinal vascular endothelial cells and retinal pigment epithelial cells, resulting in reduced VEGF production and subsequent microvascular density loss.

Our findings align with several previous studies demonstrating reduced vessel density and retinal thinning in highly myopic eyes. Shimada et al. reported lower retinal blood flow in high myopia patients, which was attributed to narrowed retinal vessel diameter and subsequent ischemia and chorioretinal atrophy [37]. Al-Sheikh et al. observed decreased retinal capillary microvasculature density and larger flow deficit regions in highly myopic eyes, consistent with our findings of reduced vessel density in the perifoveal and parafoveal regions [38]. Fan et al. similarly reported a reduction in macular vascular density with increasing myopia severity, reinforcing the association between axial elongation and microvascular remodeling [39].

Lin et al. found that the rate of CD reduction was faster in the outer ring than in the inner ring in highly myopic eyes compared to normal eyes [40]. This may be explained by the absence of large blood vessels and optic nerve fibers in the DCP, making it less resistant to excessive axial elongation of the eyeball [41]. He et al. identified a significant correlation between deep parafoveal vessel density and axial length, tilted disc ratio, and macular retinal thickness [42]. Structural elongation of the eyeball may mechanically stretch the retinal tissue and tilt the optic disc as axial elongation progresses, leading to a decrease in parafoveal retinal microvascular density. The mechanical stretching of the retinal tissue may compromise the microvascular network, reducing perfusion capacity and potentially impairing metabolic support of the retina. Furthermore, the tilting of the optic disc may cause asymmetry in the distribution of retinal nerve fibers, further exacerbating the reduction in vascular density. These structural changes may contribute to the progression of myopia-related complications, including choroidal thinning and retinal atrophy, which have been associated with worsening visual acuity and increased risk of pathological myopia.

The clinical relevance of these findings lies in the potential for early identification of microvascular changes as a predictive marker for myopia progression. Monitoring DVD and RT at the quadrant level could provide an early warning of structural and vascular remodeling in highly myopic eyes. The regional specificity of these changes suggests that targeted therapeutic strategies—such as localized pharmacologic interventions to improve retinal perfusion or biomechanical treatments to limit axial elongation—could mitigate the progression of myopia-related complications. Identifying early quadrant-specific vascular changes could enable more precise and individualized treatment strategies, potentially improving long-term visual outcomes in highly myopic patients [43,44].

These findings underscore the importance of OCTA-derived metrics in assessing myopia progression. Early detection of microvascular and structural changes could enable more effective management of high myopia, reducing the risk of irreversible vision loss and improving the quality of life for affected individuals.

This study has several limitations that should be acknowledged. First, the sample size of 260 eyes, while sufficient for detecting statistically significant differences, may limit the generalizability of the findings to broader populations with different demographic characteristics. The study included participants from a single clinical center, which may introduce selection bias and limit the applicability of the results to other populations with different genetic, environmental, or lifestyle factors. Furthermore, the sample was primarily composed of adults aged 18 to 60 years, excluding younger patients in whom myopia progression may follow different patterns and mechanisms. Second, the cross-sectional design prevents the establishment of a direct causal relationship between axial elongation and vascular changes. While significant correlations between vessel density, retinal thickness, and myopia severity were observed, a longitudinal design would be necessary to determine whether reductions in vessel density precede or follow structural retinal changes. Third, although OCTA provides high-resolution imaging of retinal microvasculature, it has inherent technical limitations. OCTA measures blood flow indirectly by detecting changes in the reflectance pattern of erythrocytes; however, it does not provide direct measurements of absolute blood flow. Motion correction algorithms were applied to minimize artifacts, but small misalignments during acquisition may have introduced noise into the data. Differences in fixation stability between myopic and emmetropic eyes may also have contributed to variability in image quality. Fourth, the study focused on macular microvasculature and did not assess peripheral retinal changes. Previous research has suggested that vascular remodeling in high myopia extends to the peripheral retina, where larger non-perfused zones and capillary dropout have been reported. The exclusive focus on the macular region may, therefore, underestimate the full extent of microvascular alterations in high myopia. Finally, potential confounding factors, such as age, gender, and baseline axial length, were adjusted for in the analysis; however, other unmeasured variables, such as systemic vascular health and genetic predisposition, may have influenced the results. A more comprehensive evaluation of these factors in future studies would help clarify the complex relationship between vascular remodeling and myopia progression [45,46].

Future research should focus on conducting large-scale longitudinal studies to establish the temporal relationship between vascular and structural changes in myopia progression. Clarifying whether reductions in vessel density precede retinal thinning or occur as a consequence of axial elongation would provide valuable insight into the sequence of disease progression. Expanding the analysis to include peripheral retinal microvasculature using wide-field OCTA would provide a more comprehensive assessment of myopia-related vascular remodeling. Exploring choroidal vascular density and thickness in combination with retinal vessel density may offer new insights into the underlying mechanisms of myopia progression. Identifying additional biomarkers for myopia progression remains an important goal. While vessel density and retinal thickness are promising indicators, other parameters such as foveal avascular zone (FAZ) size, choroidal vascular index, and retinal nerve fiber layer thickness could provide complementary information. Multimodal imaging approaches combining OCTA with structural OCT and fundus photography could enhance predictive models for myopia progression and response to treatment. Further investigation into the molecular mechanisms underlying vascular remodeling in myopia, particularly the role of VEGF and other signaling molecules, could reveal new therapeutic targets. Assessing the impact of pharmacologic agents, such as anti-VEGF therapies or drugs that enhance retinal perfusion, could provide novel treatment strategies. Finally, evaluating the effects of current myopia treatments, including low-dose atropine, orthokeratology, and peripheral defocus contact lenses, on retinal vessel density and structure could improve understanding of their mechanisms and long-term efficacy. Integrating vascular, structural, and systemic markers could lead to more precise and individualized approaches to managing high myopia and its complications.

In conclusion, this study utilized OCTA to comprehensively assess and quantify blood perfusion and retinal thickness (RT) across different degrees of myopia from a quadrant-based perspective. Our findings revealed significant alterations in the retinal vasculature and microstructure, with RT changes being more pronounced in the early stages of myopia and vascular density (VD) alterations becoming more evident in high myopia. The reduction in deep vessel density (DVD) across specific quadrants underscores the susceptibility of the deep capillary plexus to axial elongation and structural remodeling. These findings highlight the complex relationship between retinal microvascular and structural changes and the progression of myopia. This study identifies DVD and RT as promising and sensitive biomarkers for monitoring myopia progression, facilitating early diagnosis, and guiding therapeutic interventions. The quadrant-specific changes observed in vessel density and retinal thickness suggest that targeted treatments aimed at preserving deep retinal perfusion could mitigate myopia-related complications and improve visual outcomes. The ability to monitor these changes with OCTA could enable more personalized and timely interventions, improving the overall management of myopia. Future research should focus on conducting longitudinal studies to confirm the temporal relationship between vascular and structural changes in myopia progression. Additionally, integrating wide-field OCTA imaging and molecular analysis could enhance the understanding of the underlying mechanisms driving microvascular remodeling in myopia. By refining diagnostic criteria and developing targeted therapeutic approaches based on these findings, clinicians may be able to slow the progression of high myopia and reduce the risk of long-term visual impairment.

## Figures and Tables

**Table 1:** Superficial vessel density in the four diagnostic groups.

	EM	LM	MM	HM	EM-LM	EM-MM	EM-HM	LM-MM	LM-HM	MM-HM
Whole	48.12 ± 2.21	47.34 ± 1.27	47.21 ± 2.45	47.22 ± 4.22	ns	ns	ns	ns	ns	ns
Fovea	29.33 ± 4.54	28.56 ± 1.45	28.54 ± 8.21	27.78 ± 3.66	ns	ns	ns	ns	ns	ns
Parafovea	51.43 ± 6.86	50.34 ± 5.97	49.44 ± 3.91	48.21 ± 4.48	ns	ns	ns	ns	ns	ns
Paratemporal	50.35 ± 3.98	50.76 ± 2.32	50.55 ± 4.99	50.22 ± 6.11	ns	ns	ns	ns	ns	ns
Parasuperior	50.72 ± 5.54	50.55 ± 6.45	49.21 ± 3.65	48.56 ± 3.28	ns	ns	ns	ns	ns	ns
Paranasal	50.65 ± 2.23	50.21 ± 6.23	49.43 ± 5.43	48.45 ± 6.28	ns	0.017	0.001	ns	ns	ns
Parainferior	48.91 ± 7.21	49.23 ± 5.44	49.01 ± 5.82	48.87 ± 6.32	ns	ns	ns	ns	ns	ns
Perifovea	45.67 ± 2.02	45.21 ± 3.89	44.51 ± 3.54	44.27 ± 4.32	ns	ns	ns	ns	ns	ns
Peritemporal	46.21 ± 3.33	45.92 ± 4.44	45.33 ± 6.07	44.23 ± 2.28	ns	0.006	0.001	ns	0.004	0.032
Perisuperior	47.45 ± 6.56	46.69 ± 4.21	46.21 ± 2.89	45.55 ± 5.33	ns	0.005	0.001	ns	0.027	ns
Perinasal	49.83 ± 8.21	49.44 ± 6.45	48.87 ± 4.77	48.56 ± 5.55	ns	ns	ns	ns	ns	ns
Peri-inferior	48.32 ± 3.91	47.89 ± 8.21	47.21 ± 7.22	46.67 ± 3.79	ns	0.03	0.008	ns	0.004	0.012

EM—emmetropia; LM—lowmyopia; MM—moderate myopia; HM—high myopia; ns—not significant. Numbers appear as mean ± standard deviation for normally-distributed variables. Normally distributed data were analyzed usingthe one-way ANOVA and nonnormal data analysis using the Kruskal–Wallis test.

**Table 2:** Deep vessel density in the four diagnostic groups.

	EM	LM	MM	HM	EM-LM	EM-MM	EM-HM	LM-MM	LM-HM	MM-HM
Whole	49.21 ± 3.21	48.65 ± 1.22	48.33 ± 2.33	47.41 ± 3.22	ns	0.03	0.001	ns	0.003	ns
Fovea	31.21 ± 2.89	30.45 ± 3.33	30.87 ± 2.62	30.76 ± 4.34	ns	ns	ns	ns	ns	ns
Parafovea	51.21 ± 7.56	50.87 ± 1.33	50.45 ± 1.35	50.35 ± 6.67	ns	ns	ns	ns	ns	ns
Paratemporal	50.89 ± 5.54	50.67 ± 5.51	50.32 ± 4.44	50.56 ± 8.22	ns	ns	ns	ns	ns	ns
Parasuperior	51.32 ± 3.78	51.11 ± 4.98	50.89 ± 6.89	50.78 ± 7.33	ns	ns	ns	ns	ns	ns
Paranasal	53.98 ± 4.45	52.61 ± 3.33	51.34 ± 5.57	49.33 ± 6.77	ns	0.012	0.004	0.022	0.003	ns
Parainferior	51.87 ± 8.21	51.45 ± 6.32	50.67 ± 3.29	49.23 ± 7.23	ns	0.023	0.006	ns	ns	0.032
Perifovea	50.23 ± 2.46	49.67 ± 6.72	48.89 ± 6.53	47.33 ± 3.45	ns	0.017	0.002	ns	0.007	ns
Peritemporal	50.32 ± 5.32	48.21 ± 3.38	47.77 ± 2.87	46.89 ± 2.32	ns	0.009	0.002	0.025	0.003	0.026
Perisuperior	50.78 ± 7.56	49.21 ± 4.44	48.01 ± 8.32	47.11 ± 3.45	ns	0.011	0.003	0.034	0.009	ns
Perinasal	50.97 ± 6.33	49.56 ± 8.22	48.85 ± 6.33	47.65 ± 6.61	ns	0.021	0.008	ns	ns	ns
Peri-inferior	49.21 ± 5.55	48.45 ± 3.44	47.55 ± 5.22	45.78 ± 3.39	ns	0.009	0.002	ns	0.008	0.022

EM—emmetropia; LM—lowmyopia; MM—moderate myopia; HM—high myopia; ns—not significant. Numbers appear as mean ± standard deviation for normally-distributed variables. Normally distributed data were analyzed using the one-way ANOVA and nonnormal data analysis using the Kruskal–Wallis test.

**Table 3:** Thickness (µm) of retina superficial in the four diagnostic groups.

	EM	LM	MM	HM	EM-LM	EM-MM	EM-HM	LM-MM	LM-HM	MM-HM
Whole	303.23 ± 10.12	299.45 ± 13.13	295.32 ± 16.28	291.34 ± 15.72	ns	ns	<0.001	ns	0.023	ns
Fovea	255.67 ± 13.26	250.32 ± 11.82	247.11 ± 14.96	243.34 ± 12.95	ns	<0.001	<0.001	ns	<0.001	ns
Parafovea	323.45 ± 14.23	319.87 ± 17.34	315.56 ± 10.18	310.98 ± 10.54	ns	0.007	<0.001	ns	0.002	ns
Paratemporal	315.22 ± 11.21	311.12 ± 14.82	309.43 ± 12.15	306.12 ± 11.71	ns	0.027	<0.001	ns	0.014	ns
Parasuperior	332.23 ± 15.53	325.25 ± 15.23	320.38 ± 13.45	316.32 ± 10.65	0.002	<0.001	<0.001	0.032	0.003	ns
Paranasal	328.75 ± 11.78	323.76 ± 10.92	318.39 ± 15.23	308.18 ± 16.04	ns	0.006	<0.001	ns	0.001	ns
Parainferior	316.32 ± 10.76	310.87 ± 16.23	306.33 ± 10.13	302.56 ± 12.36	0.023	0.008	<0.001	ns	0.032	ns
Perifovea	322.22 ± 14.92	316.43 ± 12.53	310.89 ± 10.76	304.76 ± 13.27	0.031	0.004	<0.001	0.002	0.021	ns
Peritemporal	287.65 ± 15.13	275.43 ± 11.62	260.32 ± 11.32	264.65 ± 14.92	0.012	<0.001	<0.001	0.001	0.008	ns
Perisuperior	278.43 ± 13.62	273.32 ± 13.56	271.28 ± 13.78	266.23 ± 15.07	0.027	0.032	<0.001	ns	0.007	ns
Perinasal	294.32 ± 14.03	287.32 ± 12.69	282.28 ± 14.63	277.56 ± 10.91	0.033	0.007	<0.001	0.022	0.025	ns
Peri-inferior	301.45 ± 11.01	290.33 ± 10.65	282.34 ± 16.45	278.45 ± 12.26	0.007	<0.001	<0.001	0.012	<0.001	ns

EM—emmetropia; LM—lowmyopia; MM—moderate myopia; HM—high myopia; ns—not significant. Numbers appear as mean ± standard deviation for normally-distributed variables. Normally distributed data were analyzed using the one-way ANOVA and nonnormal data analysis using the Kruskal–Wallis test.

**Table 4:** Thickness (µm) of Retina Deep in the four diagnostic groups.

	EM	LM	MM	HM	EM-LM	EM-MM	EM-HM	LM-MM	LM-HM	MM-HM
Whole	324 ± 12.13	318 ± 13.43	305 ± 15.15	294 ± 11.44	ns	<0.001	<0.001	0.023	<0.001	ns
Fovea	334 ± 13.42	332 ± 13.78	325 ± 17.06	322 ± 12.23	ns	ns	0.014	0.021	ns	ns
Parafovea	310 ± 15.87	305 ± 15.62	290 ± 11.86	285 ± 13.72	ns	0.031	0.005	ns	0.007	ns
Paratemporal	308 ± 10.35	300 ± 16.34	280 ± 16.67	270 ± 16.43	ns	<0.001	<0.001	0.023	0.012	0.022
Parasuperior	322 ± 11.56	312 ± 14.54	280 ± 11.12	278 ± 15.03	0.022	<0.001	<0.001	0.007	<0.001	ns
Paranasal	303 ± 10.98	295 ± 18.67	292 ± 14.18	290 ± 10.82	0.025	0.007	ns	ns	ns	ns
Parainferior	315 ± 14.15	310 ± 15.06	303 ± 16.09	295 ± 12.49	ns	0.03	0.005	0.032	0.007	ns
Perifovea	303 ± 12.43	302 ± 10.89	278 ± 10.51	274 ± 11.92	ns	0.006	<0.001	0.007	<0.001	ns
Peritemporal	310 ± 14.56	306 ± 12.23	285 ± 12.63	282 ± 12.23	ns	0.007	<0.001	0.006	<0.001	ns
Perisuperior	315 ± 15.89	310 ± 12.76	282 ± 13.36	278 ± 15.82	ns	0.003	<0.001	0.004	<0.001	ns
Perinasal	320 ± 11.31	303 ± 13.41	290 ± 16.48	273 ± 13.35	0.013	<0.001	<0.001	ns	<0.001	0.004
Peri-inferior	323 ± 17.34	301 ± 10.91	292 ± 11.72	275 ± 12.07	0.007	<0.001	<0.001	ns	<0.001	0.012

EM—emmetropia; LM—lowmyopia; MM—moderate myopia; HM—high myopia; ns—not significant. Numbers appear as mean ± standard deviation for normally-distributed variables. Normally distributed data were analyzed using one-way ANOVA and nonnormal data analysis using the Kruskal–Wallis test.

## Data Availability

The data that support the findings of this study are available on request from the corresponding author.
